# Availability and Distribution of Emergency Obstetric Care Services in Karnataka State, South India: Access and Equity Considerations

**DOI:** 10.1371/journal.pone.0064126

**Published:** 2013-05-22

**Authors:** Prem K. Mony, Jayanna Krishnamurthy, Annamma Thomas, Kiruba Sankar, B. M. Ramesh, Stephen Moses, James Blanchard, Lisa Avery

**Affiliations:** 1 Division of Epidemiology & Population Health, St John's Research Institute, Bangalore, India; 2 Karnataka Health Promotion Trust, Bangalore, India; 3 Department of Community Health Sciences, University of Manitoba, Winnipeg, Canada; 4 Department of Obstetrics & Gynaecology, St John's Medical College Hospital, Bangalore, India; Tehran University of Medical Sciences, Iran (Islamic Republic Of)

## Abstract

**Background:**

As part of efforts to reduce maternal deaths in Karnataka state, India, there has been a concerted effort to increase institutional deliveries. However, little is known about the quality of care in these healthcare facilities. We investigated the availability and distribution of emergency obstetric care (EmOC) services in eight northern districts of Karnataka state in south India.

**Methods & Findings:**

We undertook a cross-sectional study of 444 government and 422 private health facilities, functional 24-hours-a-day 7-days-a-week. EmOC availability and distribution were evaluated for 8 districts and 42 taluks (sub-districts) during the year 2010, based on a combination of self-reporting, record review and direct observation. Overall, the availability of EmOC services at the sub-state level [EmOC = 5.9/500,000; comprehensive EmOC (CEmOC) = 4.5/500,000 and basic EmOC (BEmOC) = 1.4/500,000] was seen to meet the benchmark. These services however were largely located in the private sector (90% of CEmOC and 70% of BemOC facilities). Thirty six percent of private facilities and six percent of government facilities were EmOC centres. Although half of eight districts had a sufficient number of EmOC facilities and all eight districts had a sufficient number of CEmOC facilities, only two-fifths of the 42 taluks had a sufficient number of EmOC facilities. With the private facilities being largely located in select towns only, the ‘non-headquarter’ taluks and ‘backward’ taluks suffered from a marked lack of coverage of these services. Spatial mapping further helped identify the clustering of a large number of contiguous taluks without adequate government EmOC facilities in northeastern Karnataka.

**Conclusions:**

In conclusion, disaggregating information on emergency obstetric care service availability at district and subdistrict levels is critical for health policy and planning in the Indian setting. Reducing maternal deaths will require greater attention by the government in addressing inequities in the distribution of emergency obstetric care services.

## Introduction

India accounts for 17% of the world's population and contributes to 19% of global maternal deaths. It is not on track to achieving Millennium Development Goal 5 by the year 2015, pointing to the need for urgency in addressing this unfinished agenda in women's health [1].

Despite substantial improvements over the last decade, accelerated progress in India is needed for achievement of global goals. Since tracking progress in reducing maternal mortality is not easy for resource-constrained countries, the United Nations Population Fund (UNFPA), UNICEF and the World Health Organization (WHO) have proposed a proxy indicator – the monitoring of Emergency Obstetric Care (EmOC) services for complications of pregnancy and childbirth [2]. Birthing facilities are to be monitored on their ability to provide two distinctive sets of services. Those that provide six life-saving services or ‘signal functions’ (parenteral antibiotics, oxytocics, and anticonvulsants; assisted vaginal delivery; manual removal of placenta; and removal of retained products) are defined as basic EmOC (BEmOC) facilities. Those that provide these six signal functions plus Caesarean delivery and blood transfusion are categorized as comprehensive EmOC (CEmOC) facilities. Guidelines relating to the availability and distribution of EmOC services at the population-level have been proposed as benchmarks of care: it is recommended that at least five EmOC facilities, with one of them being CEmOC, are available per 500,000 population; and that they be geographically distributed across all subnational areas [2].

Maternal mortality ratio (MMR) in India for the period 2007–09 was 212 per 100,000, with values ranging from 81 to 390 across different states [3]. Since 2005, the federal government of India has launched the National Rural Health Mission (NRHM), a flagship programme committed to increasing public spending on health from 0.9% to 2–3% of GDP. This has resulted in a substantial 30% increase (from 41% to 54%) in institutional deliveries within a few years across the country [4]–[5]. The next level of investment is to follow-up on building staff capacity, improving quality of care processes and strengthening of the 3-tier health system in the country [4]. The aim of our investigation was to study the availability and distribution of emergency obstetric care services in eight districts of northern Karnataka.

## Materials and Methods

### Study setting

The estimate (95% confidence interval) of maternal mortality ratio (MMR) for Karnataka state during the period 2007–09 was 178 (124–233) per 100,000 live-births. The present study was conducted in north Karnataka, one of four agro-economic divisions of the state and one of 56 such divisions in the country [6]. This region's health indicators for the year 2010 were average for India: birth rate = 23.4 (ranked 23rd out of 56 regions with range = 13.2–32.4); death rate = 8.5 (ranked 43rd out of 56 regions with range = 4.6–11.5) and infant mortality rate = 45 (ranked 35th out of 56 regions with range = 13–83) [3]. The eight districts (Bagalkot, Koppal, Bijapur, Bellary, Raichur, Gulbarga, Yadgir & Bidar) of northern Karnataka subdivided into 42 taluks (administrative sub-districts) together had a population of 15.1 million (mid-year 2010), comprising 25% of the state's population. Female literacy rate was 42%; urbanization was 25%; and scheduled castes and tribes comprised 39% of the population in this region. Compared to the rest of the state, this region had worse-off health indicators (50% higher crude birth rate, 23.4 vs 15.6; and 35% higher infant mortality rate, 45 vs 33) [3]. As part of a 5-year project (2009–2014) to provide technical support to the Government of Karnataka for improving maternal, neonatal and child health (MNCH) care in this relatively under-developed region in the state, we carried out a baseline situational analysis of 3005 healthcare facilities in 2010. This covered assessment of: health infrastructure; health manpower; availability of drugs, equipment and supplies; population coverage of health facilities; health financing; information systems; and provision of health care services (in antenatal, intranatal, postnatal, neonatal and child health domains).

### Study design

A Needs Assessment Survey using a cross-sectional epidemiologic study design was undertaken during June–October 2010.

### Study population

There were a total of 3005 (2515 government and 490 private) health facilities in the region. Eligible study population were all “24/7” health facilities (functioning round-the-clock 7-days a week) in the government (n = 444) and private (n = 490) sectors. In the government sector, 73 ‘non-24/7’ primary health centres (PHCs) and 1998 subcentres that were not functioning round-the-clock were not included in this analysis. Thus in the government sector, there were eight district hospitals, 34 taluk hospitals, 69 community health centres (CHCs sub-categorized into 29 First Referral Units/FRU-CHCs and 40 non-FRU CHCs) and 333 “24/7” primary health centres (PHCs). In the private sector, “24/7” hospitals were categorized into groups based on number of beds per hospital, equivalent to that seen in the government sector: 1–6 beds (n = 114), 7–30 beds (n = 226), 31–50 beds (n = 31) and >50 beds (n = 11); for 40 hospitals, the bed-number was recorded as <30 but the exact number could not be ascertained. One ‘24/7’ PHC and 68 private hospitals that refused to provide relevant information were considered ‘non-responders’ and excluded from the analysis. Thus a total of 443 government and 422 private hospitals were included in the analysis.

### Study instrument, study personnel and approvals

Within the study questionnaire, there was a module on emergency obstetric care that was designed to collect information on the ability of the facility to provide the eight signal functions. The study investigators trained 20 research assistants over a period of 10 days in May 2010 to be the study interviewers. They visited all health facilities subsequently and obtained information on signal function performance through face-to-face interviews with the facility staff (medical officer, nurse, pharmacist, etc.). This self-reported information was verified by a combination of review of facility records and direct observation. Direct observation was carried out for a core set of infrastructure, drugs and supplies (with no ‘stock-out’ in the last 3 months) required to perform the signal function [7]. The following were considered mandatory: (i) any uterotonic (oxytocin/ergometrine), (ii) magnesium sulphate, (iii) functional obstetric forceps or vacuum suction apparatus, (iv) functional manual vacuum aspiration (MVA) syringe or dilatation & curettage (D&C) set, (v) a functional Caesarean section (C-section) kit, and (vi) an operation theatre where C-sections were conducted in the previous three months. Others such as antibiotics, anesthetics, blood bank/storage facility etc. were considered not necessary to be present in the health facility at the time of the study since they were frequently sourced at the time of need in various facilities.

### Ethics statement

Clearances were obtained from government authorities (Mission Director of National Rural Health Mission for Karnataka, and Project Director of Reproductive & Child Health, Department of Health, Government of Karnataka) and professional organizations (The Federation of Obstetric and Gynaecological Societies of India). Written informed consent was provided by the head of each facility. Institutional ethics approvals for this baseline assessment were obtained from St John's Medical College, Bangalore, India and the University of Manitoba, Winnipeg, Canada.

### Statistical and geographic analysis

The population of the study districts and taluks were calculated as post-censal estimates for the year 2010, from the base 2001 Census data using the geometric progression method [8]. The densities of emergency obstetric care facilities – basic (BEmOC), comprehensive (CEmOC) and total (EmOC) – were calculated per 500,000 population as per standard definitions (WHO 2009) using SAS v9.1 (SAS Institute, Cary, NC, USA). Simple descriptive analysis using ratios was undertaken; for comparative analysis, t-tests were used since they were independent samples, with a p-value<0.05 considered as significant. Since the average population of a district was 1.8 million and that of a taluk was about 350,000, availability of EmOC, BEmOC and CEmOC were all calculated at the district level while only EmOC and BEmOC were calculated at the taluk-level.

To study these indicators at the sub-district level, taluks were categorized as ‘HQ taluks’ (where the district headquarters town with the local government offices and businesses was located) or as ‘non-HQ taluks’. Taluks were also categorized as ‘most backward taluks’ or ‘non-backward taluks’ based on the classification of the Karnatakata state High Power Committee for Redressal of Regional Imbalances (HPC-FRRI) [9] which had assessed the level of development of all taluks in Karnataka state on the basis of 35 socio-economic indicators including agriculture, industry, economic/social/technical infrastructure, etc. Taking the state average of development of these indicators compiled into a single composite index as the benchmark equal to one, taluks were subclassified as ‘most backward’ (with values 0.52 to 0.79) and ‘non-backward’ (with values ≥0.80). This served to identify the relative development of a taluk on these 35 indicators as compared to the state average.

Thematic mapping with simple choropleth maps (regional statistical maps) was undertaken for visualization of the distribution of EmOC services [10]. All geographical analyses and cartographic presentation were done using ArcGIS 9.3.1 (© 2009, ESRI, Redlands, CA).

## Results

The distribution of the 444 public and 422 private health facilities in the 42 taluks of the eight districts is shown in Table 1. Overall, there were 31 health facilities (17 government and 14 private) per 500,000 population. Among districts, the number of health facilities per 500,000 ranged from 25.3 in Raichur to 43.3 in Bagalkot; among taluks, it ranged from 18.0 in Kudligi taluk (Bellary district) to 52 in Bagalkot taluk (Bagalkot district). The overall ratio of government∶private health facilities was 1.2∶1.0 in northern Karnataka; this varied from 3∶1 (Yadgir district) to a reversal of 1.0∶1.8 (Bagalkot district).

**Table 1:** Distribution of public and private maternity hospitals by taluk in the eight districts, 2010.

District (Population)	Taluk	Number of Hospitals							
		Public sector				Private sector		Total Number	
		DH*	TH*	CHC*	PHC*	≤30 beds	>30 beds	Taluk	District
Bagalkot	Bagalkot	1		1	5	18	3	29	160
(1,848,941)	Badami		1	2	8	7	1	20	
	Bilgi		1	1	2	6	0	10	
	Hungund		1	1	10	9	1	25	
	Jamkhandi		1	1	6	33	2	44	
	Mudhol		1	1	8	21	1	32	
Koppal	Koppal	1		2	11	7	1	22	78
(1,370,023)	Gangawati		1	3	9	10	3	27	
	Kushtagi		1	1	7	3	0	13	
	Yelburga		1	3	11	0	0	16	
Bijapur	Bijapur	1		0	8	37	6	56	142
(2,134,790)	B. Bagevadi		1	1	8	7	0	23	
	Indi		1	2	9	10	0	25	
	Muddebihal		1	3	5	8	0	20	
	Sindgi		1	3	6	6	0	18	
Bellary	Bellary	1		1	11	25	7	46	131
(2,476,587)	Hadagalli		1	1	9	0	0	12	
	H.Bommanahalli		1	1	7	3	0	13	
	Hospet		1	0	7	13	2	23	
	Kudligi		1	3	7	1	0	12	
	Sandur		1	1	4	3	1	10	
	Siruguppa		1	1	7	6	0	15	
Raichur	Raichur	1		1	8	14	7	31	96
(1,897,372)	Devadurga		1	2	6	1	0	10	
	Lingsugur		1	2	12	5	1	21	
	Manvi		1	1	9	5	0	16	
	Sindhnur		1	0	11	5	1	18	
Yadgir	Yadgir	1		3	11	8	0	28	68
(1,148,788)	Shahpur		1	2	10	7	0	21	
	Shorapur		1	1	9	2	0	18	
Gulbarga	Gulbarga	1		0	9	42	4	61	172
(2,522,079)	Afzalpur		1	3	7	3	0	16	
	Aland		1	2	8	3	0	20	
	Chincholi		1	2	9	3	0	16	
	Chitapur		1	5	8	4	0	24	
	Jewargi		1	2	10	6	0	20	
	Sedam		1	2	6	4	0	15	
Bidar	Bidar	1		0	7	14	2	24	93
(1,678,599)	Aurad		1	2	6	2	0	14	
	Bhasavakalyan		1	2	8	7	0	20	
	Bhalki		1	1	7	5	0	17	
	Homnabad		1	3	6	5	1	18	
Total		8	34	69	332	380	42	866	
(15,077,179)		433				422			

* DH = District hospital; TH = Taluk hospital; CHC = Community health centre; PHC = primary health centre.

There were nearly six times the number of private EmOC centres (n = 151) as there were government EmOC centres (n = 27) in the study area. For CEmOC facilities, the number of private centres (n = 121) was 8-fold higher than the number in the government sector (n = 15); and it was more than double the number for BEmOC facilities (private = 30 vs. government = 12).

For every BEmOC facility, there were three CEmOC facilities; in the private sector, there were nearly 4 CEmOC facilities for every BEmOC facility and in the government sector, there were 1.25 BEmOC facilities for every CEmOC facility. Thus, in the private sector, 29% (151/422) of facilities were CEmOC facilities and an additional 7% (30/422) were BEmOC facilities to make a total of 36% being EmOC facilities; in the government sector however, 3.3% (15/444) were CEmOC facilities and an additional 2.7% (12/444) were BEmOC facilities to make a total of 6% EmOC facilities.

**Figure 1:**
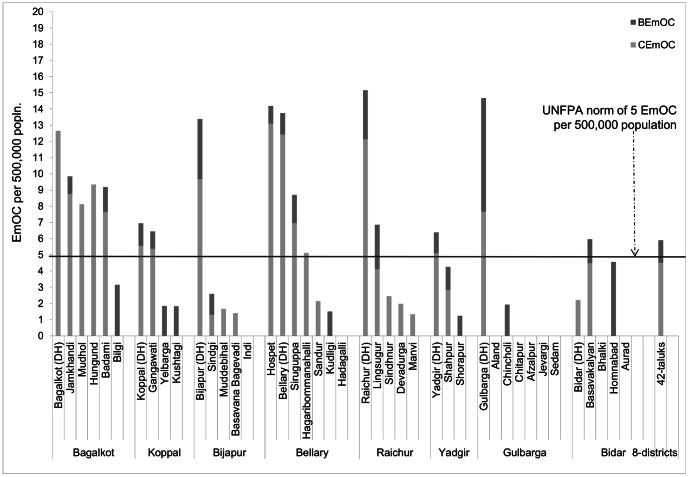
Distribution of EmOC facilities per 500,000 population by taluk, in 8 districts of northern Karnataka, 2010. EmOC = Emergency Obstetric Care; BEmOC = Basic EmOC; CEmOC = Comprehensive EmOC; DH = District headquarters taluk.

Overall, this region of northern Karnataka had sufficient number of 5.9 EmOC (including 4.5 CEmOC) facilities per 500,000 population. At the district level, half of the eight districts had sufficient number of EmOC facilities and all of them had sufficient number of CEmOC facilities. At the taluk level, only 40% (17/42) of taluks had >5 EmOC facilities per 500,000 persons. Figure 1 shows the distribution of EmOC facilities per 500,000 population in all 42 taluks in the study area. In six districts (Koppal, Bijapur, Raichur, Yadgir, Gulbarga & Bidar), less than half the taluks had an adequate number of EmOC/CEmOC facilities; in Bellary and Bagalkot districts, more than half of the taluks had adequate EmOC/CEmOC facilities. In most of the districts, the HQ taluks had a higher probability of having adequate EmOC/CEmOC facilities compared to the non-HQ taluks.

Among the 16 taluks without adequate EmOC/CEmOC facilities, it was seen that 15 (94%) did not also have the minimum desired number of 4 BEmOC facilities per 500,000 population. It was also noted that 70% of BemOC facilities were in the private sector (data not shown).

**Figure 2:**
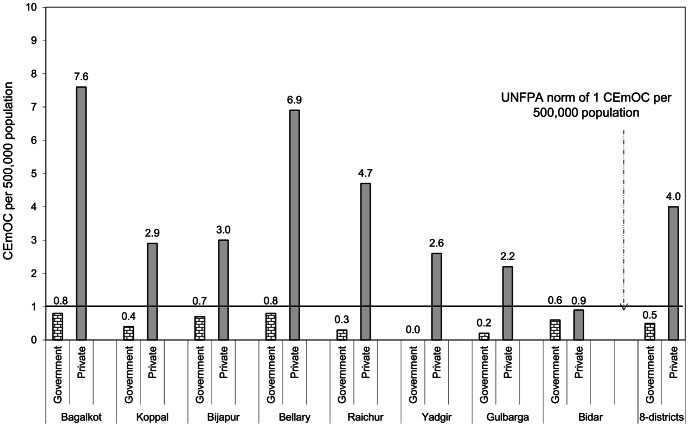
Distribution of CEmOC facilities per 500,000 population by district, northern Karnataka, 2010. CEmOC = Comprehensive Emergency Obstetric Care.

Figure 2 shows the distribution of government and private CEmOC facilities per 500,000 population in the districts. All 8 districts had sufficient number of CEmOC facilities owing to the contribution of private facilities (90%). Overall, in the government sector, northern Karnataka had only 0.5 CEmOC per 500,000 population (with all districts having <1 CEmOC/500,000 population). About 62% (26/42) of taluks had <1 CEmOC facility per 500,000 population (data not shown).

**Figure 3:**
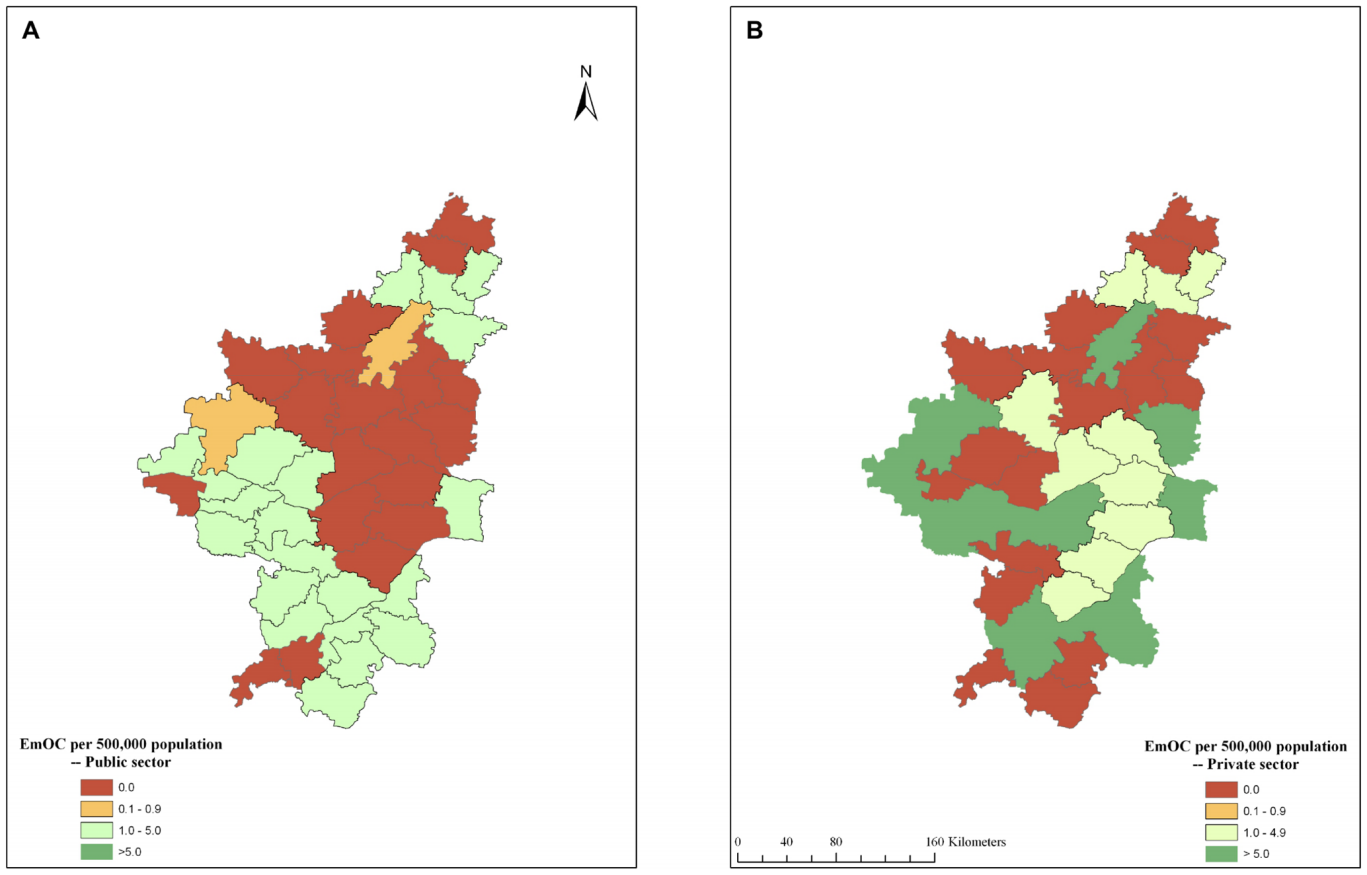
Geographic distribution of government and private EmOC facilities in 42 taluks of northern Karnataka, 2010. A. Government sector EmOC. B. Private sector EmOC. EmOC = Emergency Obstetric Care.

**Figure 4:**
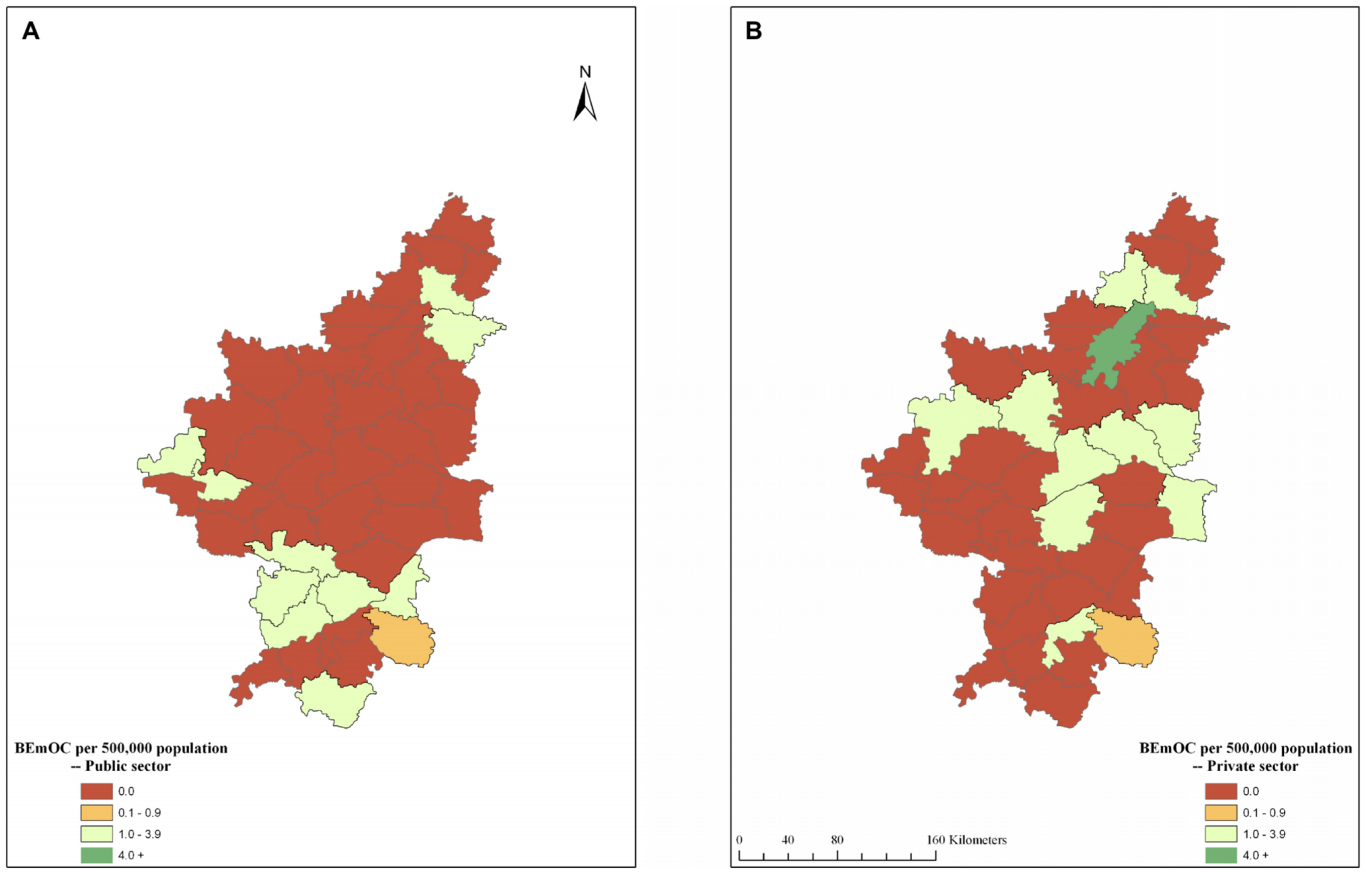
Geographic distribution of government and private BEmOC facilities in 42 taluks of northern Karnataka, 2010. A. Government sector EmOC. B. Private sector EmOC. BEmOC = Basic Emergency Obstetric Care.

The geographic distribution of EmOC facilities in the public and private sectors across the 42 taluks is shown in Figures 3(a) and (b) respectively. Similarly, the geographic distribution of BEmOC facilities in the public and private sectors across the 42 taluks is shown in Figures 4(a) and (b) respectively. None of the 42 taluks had an adequate number of EmOC or BEmOC facilities in the government sector. The private sector however, contributed substantial amount of EmOC services in 15 taluks. Within this entire region, a subset of about a dozen contiguous taluks with insufficient number of EmOC and BEmOC facilities in the government sector were spatially clustered in northeastern Karnataka across the districts of Raichur, Yadgir and Gulbarga.

The availability of EmOC facilities by type and the developmental status of taluks is depicted in Table 2. EmOC and CEmOC facilities were preferentially located in the headquarter taluks compared to the other taluks. This was largely due to a preponderance of private CEmOC facilities being sited in these headquarter taluks. There was no such preferential location of government CEmOC centres; the government BEmOC facilities were however seen more often in non-headquarter taluks than in headquarter taluks. There was no significant difference in the location of the private BEmOC facilities also.

**Table 2:** Availability of EmOC facilities per 500,000 population, by type and developmental status of taluk (2010).

Services	Taluk type†			Taluk development status#		
	HQ taluks (n = 8)	Non-HQ taluks (n = 34)	t-test (*df* = 40); p value	Most backward taluks (n = 25)	Non-backward taluks (n = 17)	t-test (*df* = 40); p value
EmOC	16.6	8.5	3.9; 0.0001*	3.3	15.0	−5.7; 0.0001*
CEmOC – total	13.1	6.8	3.5; 0.001*	1.5	12.3	−5.9; 0.0001*
BEmOC – total	3.5	1.9	1.8; 0.08	1.8	2.7	−1.1; 0.28
CEmOC – govt	0.9	0.5	1.6; 0.12	0.3	1.0	−3.2; 0.003*
CEmOC – private	12.2	3.6	3.6; 0.001*	1.1	11.3	−5.8; 0.001*
BEmOC – govt	0.2	1.0	−3.2; 0.003*	1.0	0.7	0.9; 0.38
BEmOC – private	3.3	0.8	2.2; 0.06	0.8	2.6	−1.8; 0.09

† taluk type = taluk with district headquarters (HQ taluk) & other taluks (non-HQtaluks);

# taluk developmental status according to 35 socioeconomic indicators = ‘most backward’ taluks and ‘’non-backwardtaluks;

* significant p-value<0.05.

The number of EmOC and CEmOC centres in the ‘most-backward’ taluks was much lower (one-fourth and one-sixth respectively) than in the ‘non-backward’ taluks. This was mainly because of a seven-fold higher number of private CEmOC facilities in these non-backward taluks compared to the most-backward taluks. The government CEmOC centres were also similarly preferentially located in these non-backward taluks compared to the most-backward taluks. Government and private BEmOC centres were located somewhat equally across the non-backward and the most backward taluks.

## Discussion

This is the first examination of the availability and distribution of emergency obstetric care at the district and subdistrict level in India. Overall availability of EmOC services at the population level (5.9 per 500,000) was seen to meet the benchmark in this northern region of Karnataka state in India. This is a higher ratio than has been reported from some sub-Saharan African and south Asian countries [11]–[19] but lower than that seen in a province in China (11 per 500,000) [20]. There were 4.5 per 500,000 CEmOC facilities and 1.4 per 500,000 BEmOC facilities. Although half of the districts had overall a sufficient number of EmOC facilities and all eight districts had a sufficient number of CEmOC facilities, only two-fifths of the 42 taluks had a sufficient number of EmOC facilities. The CEmOC facilities were concentrated in only a few taluks; among those taluks without an adequate number of CemOC facilities, over 90% did not have an adequate number of BEmOC facilities. The ‘non-headquarter’ taluks and ‘most backward’ taluks, in particular, suffered from a marked lack of coverage of these facilities. While inter-district inequities in distribution and availability of EmOC have been noted elsewhere, for example in Rajasthan state in northern India [11] and in Bangladesh [21], inequality at the subdistrict taluk level is a new finding of our study.

An interesting finding was that there were a greater absolute number of private EmOC facilities than government EmOC facilities in this region of southern India. We also found that proportionately, private health facilities were more likely to be EmOC facilities than were public sector facilities. Hence the private sector contributed to 89% (121/136) of the CEmOC availability and 70% (30/42) of the BEmOC availaibility in this region. Assisted vaginal deliveries [22] and parenteral administration of magnesium sulphate were the two signal functions that were missing in several health facilities, especially in the government sector, and rendering them as non-BEmOC centres; similarly, lack of blood transfusion services [23] rendered the higher-level facilities as non-CEmOC centres.

Another important finding of our study was that the presence of an acceptable number of EmOC and CEmOC facilities in several districts and taluks was due to the large number of private hospitals in the urban areas of the relatively better-off taluks. The use of Geographic Information Systems (GIS) maps further helped identify the clustering of a majority of taluks without adequate EmOC or BEmOC facilities in the government sector.

The contiguous districts of Raichur, Yadgir and Gulbarga (commonly known as ‘Hyderabad-Karnataka’) had only two government CEmOC facilities for a total of 15 taluks (equivalent to 0.2/500,000 population). This has equity implications, since private facilities can be accessed only through direct, out-of-pocket expenditures.

Our study findings point to three important recommendations. Firstly, health research examining the availability and distribution of EmOC facilities at subnational levels is a necessary first step in order to identify inequities in access to emergency obstetric care services for populations. Secondly, there is a need for translating this knowledge to policy-makers, underscoring the need to build ‘reach’ in the planning of maternal health programming. While we have used both subdistrict type and developmental status to highlight EmOC coverage because of the availability of such information in Karnataka, it is likely that other states or countries might use available information on either development status or subdistrict type to identify areas for improving EmOC coverage. Lastly, the government must take responsibility for public health sector facilities to be accessible and to be equitably distributed for the population. In our study region, the state government has already located basic emergency care centres away from the district headquarters and in the relatively backward taluks. There also needs to be a special focus on the northeastern (‘Hyderabad-Karnataka’) area. Key health system level changes will need to be effected in this regard. Restrictive policies that limit operative obstetrics and anaesthesiology to professional obstetricians and anaesthesiologists respectively, and barriers to an effective blood banking service need to be urgently addressed to improve the availability and distribution of CEmOC services. Similarly, optimal training for skilled birth attendants in instrumental delivery and management of preeclampsia/eclampsia will help increase the access to BEmOC services [24]–[28].

Our study has some limitations. Firstly, despite the algorithm used in the estimation of district and taluk populations, these post-censal projections are subject to limitations; inaccuracy increases as annual estimates become more temporally removed from the most recent census as in our case where the census 2001 occurred nine years prior to our study.

While this could theoretically alter EmOC population rates, this effect would likely be small overall given that our post-censal estimates did not differ substantially (<1%) from inter-censal estimates of populations for these districts and taluks based on the 2001 and 2011 census data.

Secondly, while we covered all government health facilities in the study area, some private health facilities were left out owing to refusal to participate in the study. If there were EmOC facilities amongst those centres, then our population-level estimate would be an underestimate. Finally, we used a combination of self-reporting, record review and direct observation of drugs/equipment/supplies to ascertain EmOC availability in all health facilities; differences in measurement methods between studies might have affected our results, as has been shown in comparisons across studies [29]–[30]. Though functionality of facilities may need to be evaluated more rigorously, our methodology is probably sufficient to identify inter-regional differences.

In summary, disaggregating information on emergency obstetric care availability at district and subdistrict levels is critical for the Indian setting. Increased investment in health infrastructure in under-served taluks, enhancement of human resources and quality improvement initiatives for adoption of obstetric care standards and protocols, could improve the geographic and financial accessibility of emergency obstetric care services for disadvantaged populations. The time for improving access and equity of emergency obstetric care services provision at district- and subdistrict-levels is now.
